# Areola Size and Jugulum Nipple Distance After Bilateral Mastectomy and Breast Reconstruction

**Published:** 2013-11-01

**Authors:** Joaquín Pérez-Guisado, Consuelo Rodríguez-Mérida, Luis F. Rioja

**Affiliations:** ^a^Service of Plastic, Aesthetic and Reconstructive Surgery, Reina Sofía University Hospital; ^b^Hospital Provincial de Cordoba, Reina Sofía University Hospital, Av. Menéndez Pidal s/n 14004, Córdoba, SPAIN

**Keywords:** areola, breast cancer, breast reconstruction, mastectomy, nipple, NAC tattooing

## Abstract

**Objective:** The combination of a single pedicle local flap with tattooing for complete nipple areola complex (NAC) reconstruction is currently the most supported method. Although many technical descriptions of NAC reconstruction exist in the medical literature, there are no data that define the ideal areola size (diameter of the areola) after bilateral mastectomy and breast reconstruction considering the previous areola size. **Methods:** This was a 3-year (2009-2012) observational, analytical, and longitudinal prospective study with 103 patients who had undergone NAC tattooing as the last process of bilateral breast reconstruction after surgery for breast cancer. Statistical differences in the areola size and the jugulum-nipple distance before mastectomy and after reconstruction were analyzed by paired Student *t* tests with a 95% confidence interval. **Results:** The jugulum-nipple distance before mastectomy was 4.23 cm larger than after bilateral reconstruction (mean jugulum-nipple distance: 23.89 cm vs 19.66 cm), and for that reason shorter (more cephalad). The areola size before mastectomy was 1.59 cm larger than the one chosen by the patient for reconstruction (mean diameter of the areola: 5.25 cm vs 3.65 cm). **Conclusions:** We conclude that, after bilateral mastectomy and reconstruction, the jugulum-nipple distance is smaller and women prefer smaller areola sizes.

Immediate breast reconstruction (IBR) has become an established procedure for women who require mastectomy. Traditionally, the nipple-areola complex (NAC) is resected during this procedure. The NAC, in turn, is a principal factor in determining esthetic outcomes after breast reconstruction, and due to its particular texture and shape, a natural-looking NAC cannot be easily reconstructed with other tissues.^1^ There are many surgical techniques available to recreate the NAC,^2^ although a combination of a single pedicle local flap with tattooing for complete NAC reconstruction is currently the most supported method,^3^ and is the one we use. The reason is that the NAC is easily reproduced, is rapid, and there is no graft. Moreover, the choice of incision method provides good tolerance. In addition, complications are rare and it is always possible to use other techniques in the case of poor results.^4^

Although many technical descriptions of NAC reconstruction exist in the medical literature, there are no data available that define the ideal areola size (diameter of the areola) after bilateral mastectomy and breast reconstruction considering the previous areola size.

## MATERIAL AND METHODS

This was a 3-year (2009-2012) observational, analytical, and longitudinal prospective study with 103 patients who had undergone NAC tattooing as the last process of bilateral breast reconstruction after surgery for breast cancer. A combination of a single pedicle local flap followed 3 to 6 months later by tattooing was performed for complete NAC reconstruction. To simplify the study analysis, we included the reconstructions of patients that were originally performed with an expander, followed by a second surgery to replace the expander with implants. The areola size before mastectomy and the jugulum-nipple distance before and after bilateral reconstruction were measured using a tape measure. The reference points for the jugulum-nipple distance were the sternal notch and the nipple. The tattoos were made between 3 to 6 months after nipple reconstruction.

After the tattooing of the NAC reconstruction, new measurements were made at the medical check-up 3 months later. The last check-up was 1 year later. Ethical approval from the hospital's ethics committee was not required for this research.

**Figure 1:**
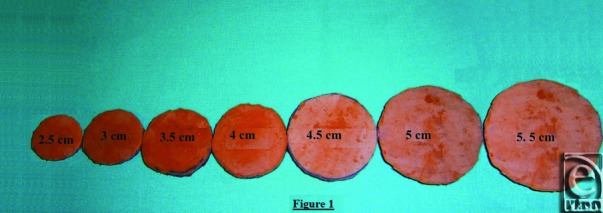
Circular sticky areola templates.

Before the NAC tattoo, the patient is shown a sample with different sizes (2.5-6 cm) of circular sticky areola templates (Fig 1). She then sticks them on her breasts and, using a mirror to see her reflection, proceeds to evaluate which sample is most appealing to her. The intended locations of these NAPs are also chosen by the patient. The nipples are placed by the plastic surgeon in the middle of the NAP location chosen by the patient. The procedure is performed by a plastic surgeon or a nurse trained in this procedure.

Statistical differences between the areola size before mastectomy and after choosing the areola template were analyzed by paired Student *t* tests using SPSS 12.0 (SPSS Inc, Chicago, IL) and are expressed as mean ± standard error of the mean with a 95% confidence interval. Before the Student *t* test, Kolmogorov-Smirnov and Shapiro-Wilk tests were used to assess normality, and the assumption of homoscedasticity was determined by the Snedecor *F* test.

## RESULTS

**Figure 2:**
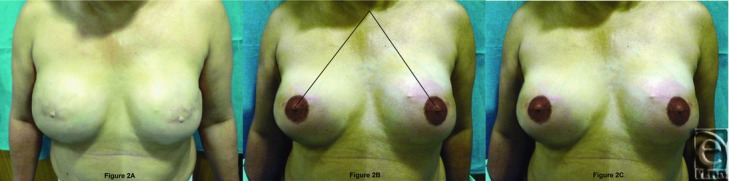
A: Results 3 months after nipple reconstruction; B: jugulum-nipple distances; C: Results 3 months after tattooing.

The mean age, weight, height, body mass index, the jugulum-nipple distances before mastectomy versus after reconstruction, areola sizes before mastectomy versus after reconstruction and tattoo follow-ups (3 months and 1 year later) of the 103 patients are shown in Table 1. Figure 2A shows the results 3 months after nipple reconstruction, Figure 2B the jugulum-nipple distances, and Figure 2C the results 3 months after tattooing.

The 2-tailed *P* values for the variables studied in the 103 patients were less than 0.001, which is considered to be highly statistically significant.

## DISCUSSION

We agree that NAC tattooing is a safe and effective technique for the restoration of the nipple-areola complex following breast reconstruction.^5^ Ideal reconstruction of the NAC requires symmetry in terms of position, size, shape, texture, pigmentation, permanent projection,^3^ and creating an inconspicuous scar.^6^

According to Hoffman and Mikell,^7^ the tendency for the pigment to fade varies, but touch-ups are often required and can be performed at any time; however, these authors did not indicate approximately what percentage of patients needed touch-ups in their study. We agree with these findings and we supplement them by reporting that 3 months after tattooing, 18.45% of the tattooings (38 tattooings) needed touch-ups, although 1 year later, none of them needed touch-ups. Nevertheless, our findings contrast those reported by Bhatty and Berry,^8^ who declared that after 2 months to 4 years of follow-up, 12.5% of the tattooings required further touch-ups.

We consider that the esthetically ideal areola size is a personal matter. There are no rules regarding esthetic opinions and there is a wide variety of naturally occurring areola sizes. However, our results demonstrate that the esthetically ideal areola size after bilateral reconstruction is a mean of 3.65 cm, which is smaller than the mean of the natural areola size before mastectomy (5.25 cm). Why do women prefer a smaller areola after bilateral mastectomy reconstruction? It is possible that a smaller jugulum-nipple distance after bilateral reconstruction plays an important role, because this is related to changes in breast size and shape after reconstruction. Nevertheless, it might also be a question of fashion or personal preference, because women may consider that an areola size of 3.5 to 4 cm is more in fashion than bigger ones.

In connection with the jugulum-nipple distance, according to Penn, the most esthetic jugulum-nipple distance is 21 cm, at which nipples should be placed at the 2 basal angles of an imaginary equilateral triangle that has its apex at the jugulum.^9^ Our results on reconstructed breasts are close to those cited by Penn (19.66 ± 0.61 cm), perhaps because the new breast size is smaller, so the patient might have chosen a smaller NAC with shorter jugulum-nipple location to be proportionate with this new breast size. However, the jugulum-nipple distance before mastectomy was 4.23 cm larger than after bilateral reconstruction (23.89 cm vs 19.66 cm) and therefore larger than the 21 cm described by Penn. We consider this is due to the fact that Penn based his idea on a small sample of young women (18- and 39 years old) and our population was older (54.38 ± 0.94 years). With increasing age and weight of the breasts, there is a proven inferior migration of the nipple and the IMF (inframammary fold), resulting in ptosis and some lateral deviation.^10^

Nevertheless, we consider as a limitation of this study that we measured the jugulum-nipple distance of the patient but we did not measured the real size of the breast before and after reconstruction, because for a real measurement of the breast size of the patient, we would also need to know the degree of ptosis of the patient before and after reconstruction.

We conclude that, after bilateral mastectomy and reconstruction, the jugulum-nipple distance is smaller and women prefer smaller areola sizes.

## Figures and Tables

**Table 1:** Measurements before bilateral mastectomy versus after reconstruction

Measurements, mean ± SEM	
Age, y	54.38 ± 0.94
Weight, kg	71.05 ± 1.12
Height, cm	158 ± 1.08
Body mass index, mean, kg/m^2^	28.46
Areola size before mastectomy, cm	5.24 ± 0.12
Areola size after reconstruction, cm	3.65 ± 0.05
Jugulum-nipple distance before mastectomy, cm	23.89 ± 0.90
Jugulum-nipple distance after reconstruction, cm	19.66 ± 0.61
*P*	<0.001
Other data, n (%)	
Patients	103 (100)
Number of NAC tattooings	206 (100)
Tattooings that needed touch-ups 3 months later	38 (18.45)
Tattooings that needed touch-ups 1 year later	0 (0)

NAC indicates nipple areola complex; SEM, standard error of the mean.
